# Radiomics in liver diseases: Current progress and future opportunities

**DOI:** 10.1111/liv.14555

**Published:** 2020-07-02

**Authors:** Jingwei Wei, Hanyu Jiang, Dongsheng Gu, Meng Niu, Fangfang Fu, Yuqi Han, Bin Song, Jie Tian

**Affiliations:** ^1^ Key Laboratory of Molecular Imaging Institute of Automation Chinese Academy of Sciences Beijing China; ^2^ Beijing Key Laboratory of Molecular Imaging Beijing China; ^3^ Department of Radiology West China Hospital Sichuan University Chengdu China; ^4^ Department of Interventional Radiology The First Affiliated Hospital of China Medical University Shenyang China; ^5^ Department of Medical Imaging Henan Provincial People’s Hospital Zhengzhou Henan China; ^6^ Department of Medical Imaging People’s Hospital of Zhengzhou University. Zhengzhou Henan China; ^7^ Beijing Advanced Innovation Center for Big Data‐Based Precision Medicine School of Medicine Beihang University Beijing China; ^8^ Engineering Research Center of Molecular and Neuro Imaging of Ministry of Education School of Life Science and Technology Xidian University Xi’an Shaanxi China

**Keywords:** data science, liver diseases, machine learning, precision medicine, radiologic technology

## Abstract

Liver diseases, a wide spectrum of pathologies from inflammation to neoplasm, have become an increasingly significant health problem worldwide. Noninvasive imaging plays a critical role in the clinical workflow of liver diseases, but conventional imaging assessment may provide limited information. Accurate detection, characterization and monitoring remain challenging. With progress in quantitative imaging analysis techniques, radiomics emerged as an efficient tool that shows promise to aid in personalized diagnosis and treatment decision‐making. Radiomics could reflect the heterogeneity of liver lesions via extracting high‐throughput and high‐dimensional features from multi‐modality imaging. Machine learning algorithms are then used to construct clinical target‐oriented imaging biomarkers to assist disease management. Here, we review the methodological process in liver disease radiomics studies in a stepwise fashion from data acquisition and curation, region of interest segmentation, liver‐specific feature extraction, to task‐oriented modelling. Furthermore, the applications of radiomics in liver diseases are outlined in aspects of diagnosis and staging, evaluation of liver tumour biological behaviours, and prognosis according to different disease type. Finally, we discuss the current limitations of radiomics in liver disease studies and explore its future opportunities.

**Abbreviations**
AFP: α‐fetoprotein
ALB: serum albumin
ALT: serum alanine aminotransferase
AST: aspartate aminotransferase
AUC: area under the curve
CA 19‐9: carbohydrate antigen 19‐9
CB: conjugated bilirubin
CNN: convolution neural network
CT: computed tomography
DL: deep learning
HBsAg: hepatitis B virus surface antigen
HCC: hepatocellular carcinoma
ICC: intrahepatic cholangiocarcinoma
MRI: magnetic resonance imaging
NASH: nonalcoholic steatohepatitis
PD‐1: anti‐programmed cell death protein
PD‐L1: anti‐programmed cell death ligand 1
PIVKA‐II: prothrombin induced by vitamin K absence‐II
PLT: platelet count
PT: prothrombin time
ROI: region of interest
SWE: shear wave elastography
TACE: transcatheter arterial chemoembolization
TB: Serum total bilirubin

**Key points**

- Radiomics as an emerging technique based on medical imaging analysis is more commonly used in liver disease studies.
- Inter‐personal heterogeneity could be revealed via extracting high‐dimensional quantitative imaging features and analysed by artificial intelligence algorithms.
- Radiomics can be applied in the diagnosis, treatment effect evaluation and prognosis prediction in liver diseases.

## 1 INTRODUCTION

Liver diseases, a wide spectrum of pathologies from inflammation to neoplasm, have become a major health problem worldwide. Noninvasive imaging plays a critical role in the characterization and monitoring of liver diseases. Conventional ultrasound, computed tomography (CT) and magnetic resonance imaging (MRI) are widely used for qualitative evaluation of liver morphology and blood supply.^1^, ^2^, ^3^ Tremendous progress is still being made in liver imaging with introduction of advanced techniques, including metabolic imaging, molecular imaging, and multi‐parametric functional MRI, etc, allowing improved evaluation of liver diseases and assisting personalized medical decision making.^4^, ^5^, ^6^

With accumulation of scalable liver imaging data, radiomics emerges as a novel radiological technique that comprehensively utilizes large‐scale medical imaging into the process of liver disease management via artificial intelligence techniques.^7^, ^8^ It enables extraction of high‐throughput quantitative imaging features beyond inspections of naked human eyes and converting encrypted medical imaging into minable numerical data.^8^ Combined with clinical, pathological, or genetic information, radiomics would assist in lesion characterization, preoperative diagnosis, treatment efficacy evaluation, as well as prognosis prediction in various clinical settings.^9^, ^10^, ^11^

Quantitative imaging traits were proved to be associated with global gene expression programmes, and could reconstruct 78% of the global gene expression profiles in liver cancer.^12^ This groundbreaking result laid a foundation and greatly encouraged researchers to explore the potential of quantitative imaging tool in preoperative genetic/pathological outcome prediction. Hence, a great deal of radiomics studies have been conducted using multi‐parametric and multi‐modality imaging in terms of liver disease diagnosis and treatment decision making.^13^, ^14^, ^15^, ^16^, ^17^, ^18^, ^19^, ^20^, ^21^, ^22^, ^23^, ^24^, ^25^, ^26^, ^27^, ^28^, ^29^, ^30^, ^31^, ^32^, ^33^, ^34^, ^35^, ^36^, ^37^, ^38^, ^39^, ^40^, ^41^, ^42^, ^43^, ^44^, ^45^, ^46^, ^47^, ^48^ In certain scenarios, this artificial intelligence‐based technique could even compete pathological gold standard, providing new ways for unsolved clinical problems in the paradigm of liver disease management.^16^ Nevertheless, it still requires further multi‐centre and prospective validation for the validity of radiomics. The interpretability and the correlation with biological/pathological underpinnings also represent substantial obstacles for the translation of artificial intelligence into real clinical practice.

**FIGURE 1:**
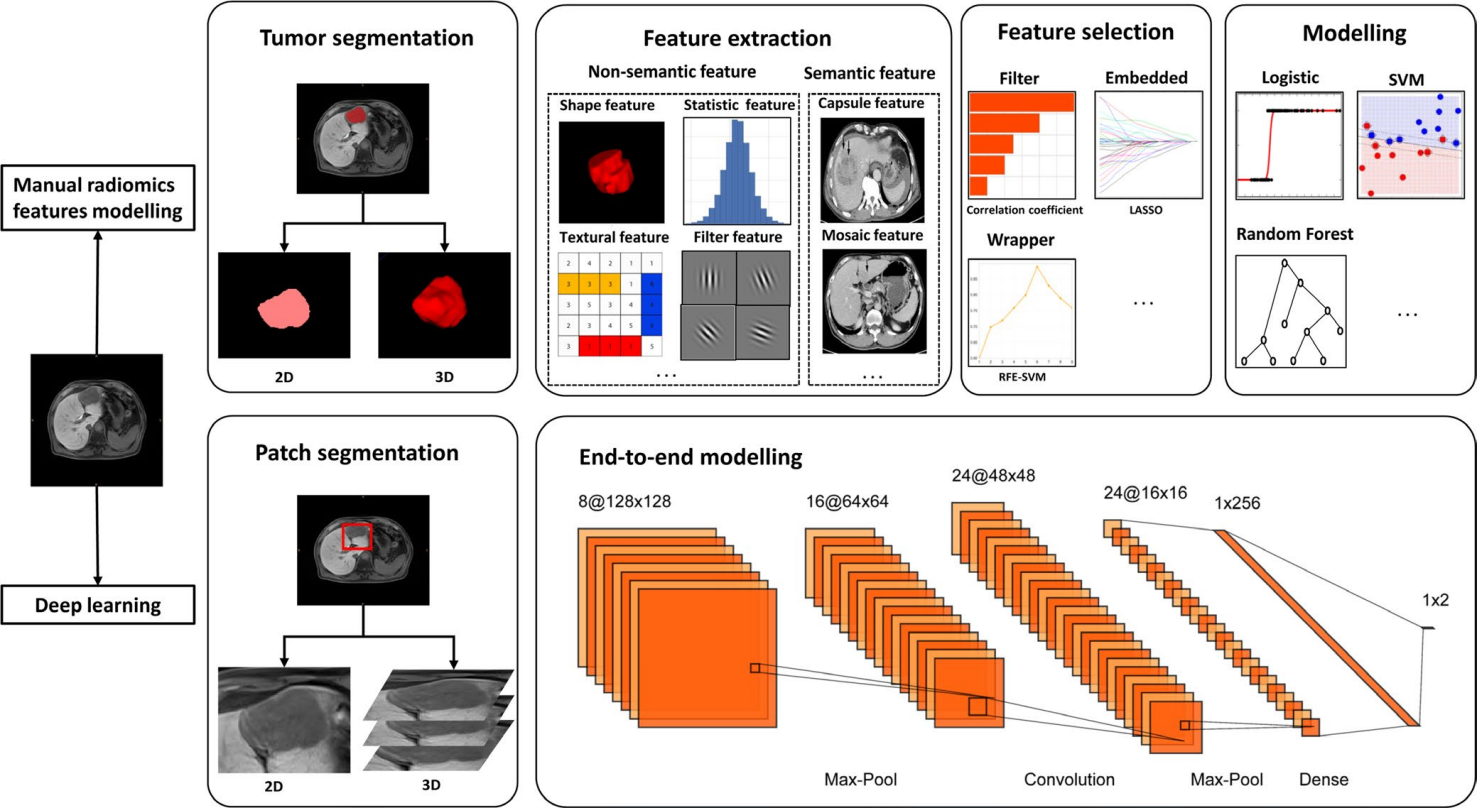
Workflow of radiomics methodological process

Here, we review the basic concepts of radiomics methodologies specific for liver studies from data acquisition, liver/lesion segmentation, feature design, to model construction (Figure 1). Meanwhile, representative clinical applications of radiomics in liver diseases regarding diagnosis, staging, evaluation of liver tumour biological behaviours, and prognosis are also within the scope of this study. Finally, we summarize the current challenges and limitation of radiomics, and explore its future directions in liver diseases.

## 2 METHODOLOGY OF RADIOMICS IN LIVER DISEASES

### 2.1 Data acquisition and curation

Data used in radiomics studies can be single‐centre or multi‐centre, and retrospective or prospective. Here, we searched PubMed (8 October 2019) for radiomics studies on liver diseases using terms (liver diseases AND radiomics), and found 36 clinical target‐oriented published work.^13^, ^14^, ^15^, ^16^, ^17^, ^18^, ^19^, ^20^, ^21^, ^22^, ^23^, ^24^, ^25^, ^26^, ^27^, ^28^, ^29^, ^30^, ^31^, ^32^, ^33^, ^34^, ^35^, ^36^, ^37^, ^38^, ^39^, ^40^, ^41^, ^42^, ^43^, ^44^, ^45^, ^46^, ^47^, ^48^ Most (33 out of 36) studies were performed on single‐centre with retrospective cohort, while only two studies were performed on multi‐centre and prospective cohort (Table 1). And the most commonly used imaging modality was CT (18 studies), followed by MRI (12 studies), positron emission tomography (PET) (two studies) and ultrasonography (US) (four studies) (Table 1).

**TABLE 1:** Summary of published radiomics studies on liver diseases

Number	Reference	Study design (retrospective/prospective, single or multi‐centre study)	No. of patients	No. and type of radiomic features	Statistical analysis (feature selection and modelling)	Imaging Modality	Clinical Characteristics
1	Zhou et al^13^	Retrospective, single‐centre study	215	300 (histogram and GLCM)	LASSO	CT	Prediction of early recurrence in HCC
2	Cozzi et al^14^	Retrospective, single‐centre study	138	35 (histogram and texture)	Cox	CT	Predict local control and survival of HCC
3	Naganawa et al^15^	Retrospective, single‐centre study	88	6 (histogram)	Logistic	CT	Prediction of nonalcoholic steatohepatitis
4	Wang et al^16^	Prospective, multi‐centre study	398	Deep learning features	DLRE	Ultrasound	Assessing liver fibrosis
5	Peng et al^17^	Retrospective, single‐centre study	304	980 (histogram, shape and texture)	LASSO	CT	Prediction of microvascular invasion
6	Reimer et al^18^	Retrospective, single‐centre study	37	6 (histogram)	Logistic	MRI	Assessment of Therapy Response to TACE
7	Akai et al^19^	Retrospective, single‐centre study	127	96 (histogram)	RSF	CT	Predicting prognosis of resected HCC
8	Li et al^20^	Retrospective, single‐centre study	144	472 (radiomics, ORF and CEMF features)	RF, SVM, DT, NN, Logistic	Ultrasound	Assessing liver fibrosis
9	Hui et al^21^	Retrospective, single‐centre study	50	290	1‐nearest neighbor	MRI	Prediction of early recurrence in HCC
10	Kim et al^22^	Retrospective, single‐centre study	88	116	LASSO, COX	CT	Predicting survival after TACE
11	Liu et al^23^	Prospective, multi‐centre study	385	20 648 (non‐texture and texture)	LASSO	CT	Noninvasively detect CSPH in cirrhosis
12	Wu et al^24^	Retrospective, single‐centre study	170	328 (non‐texture and texture)	LASSO	MRI	Predicting the grade of HCC
13	Yao et al^25^	Retrospective, single‐centre study	177	Deep learning features	KSVD + SRT+SVM	Ultrasound	Preoperative diagnosis
14	Hu et al^26^	Retrospective, single‐centre study	482	1044 histogram and texture	LASSO	Ultrasound	Prediction of microvascular invasion
15	Klaassen et al^27^	Retrospective, single‐centre study	69	370 (histogram, shape, texture)	Random forest	CT	Prediction of esophagogastric Cancer Liver Metastasis
16	Zheng et al^28^	Retrospective, single‐centre study	319	110 texture features	LASSO	CT	Preoperative Prediction of survival
17	Park et al^29^	Retrospective, single‐centre study	436	8 histogram and 35 textural features	logistic regression with elastic net regularization	MRI	Preoperative prediction of staging liver fibrosis
18	Chen et al^30^	Retrospective, single‐centre study	207	1044 radiomic features	Extremely randomized tree	MRI	Preoperative prediction of immunoscore
19	Feng et al^31^	Retrospective, single‐centre study	160	1044 radiomic features	Lasso	MRI	Preoperative prediction of microvascular invasion
20	Ma et al^32^	Retrospective, single‐centre study	157	647 (histogram, shape, texture, wavelet)	SVM	CT	Prediction of microvascular invasion
21	Shan et al^33^	Retrospective, single‐centre study	156	1044 (histogram, wavelet, texture)	LASSO	CT	Prediction of early recurrence in HCC
22	Cai et al^34^	Retrospective, single‐centre study	125	713 (intensity, texture, wavelet, shape and size)	LASSO, Logistic	CT	Prediction of Posthepatectomy Liver Failure in HCC
23	Wu et al^35^	Retrospective, single‐centre study	369	1029 (first‐order, shape, texture, high‐order)	Variance threshold, LASSO, Decision tree, Random forest, K nearest neighbors, Logistic	MR	Prediction of hepatocellular carcinoma and hepatic haemangioma
24	Xu et al^36^	Retrospective, single‐centre study	495	7260 radiomic features	Multivariable logistic regression	CT	Prediction of microvascular invasion
25	Rahmim et al^37^	Retrospective, single‐centre study	52	41 (histogram)	Univariate and multivariate	PET	Prognostic model for colorectal Liver Metastasis
26	Yuan et al^38^	Retrospective, single‐centre study	184	647 (intensity, texture, wavelet, shape and size)	MRMR, LASSO, Cox	CT	Prediction of early recurrence in HCC
27	Zhang et al^39^	Retrospective, single‐centre study	155	385 (histogram, texture)	LASSO	MR	Prediction of early recurrence in HCC
28	Zhao et al^40^	Retrospective, single‐centre study	47	396 (histogram, texture, Haralick, morphological)	Wilcoxon signed‐rank test, Logistic	MR	Prediction of early recurrence in intrahepatic cholangiocarcinoma
29	Guo et al^41^	Retrospective, single‐centre study	133	853 radiomic features	Lasso	CT	Prediction of recurrence in hcc after liver transplantation
30	Tseng et al^42^	Retrospective, single‐centre study	169	1474 radiomic features	LASSO	CT	Prediction of portal pressure and patient outcome in hypertension
31	Hectors et al^43^	Retrospective, single‐centre study	48	218 radiomic features	Binary logistic regression analysis	MRI	Prediction of immune‐oncological characteristics
32	Ni et al^44^	Retrospective, single‐centre study	206	1044 textural features	LASSO + BPNet	CT	Prediction of microvascular invasion
33	Liao et al^45^	Retrospective, single‐centre study	142	57 radiomic features	linear elastic‐net model	PET	Evaluation of Tumour‐Infiltrating CD8 + T Cells
34	Huang et al^46^	Retrospective, single‐centre study	100	First order statistical, shape, textural, and higher order statistical features	LASSO	MRI	Diagnosis of dual‐phenotype HCC
35	Shur et al^47^	Retrospective, single‐centre study	102	114 radiomic features	Multivariate cox propotional hazard modelling	CT	Improved prognostication of surgical candidates with colorectal liver metastasis
36	Jiang et al^48^	Prospective, single‐centre	211	396 radiomic features	LASSO	MRI	Diagnosis of HCC

Considering the effect of inconsistent imaging acquisition protocol and reconstruction procedure in multi‐centres via multi brand manufactories, preprocessing of the collected imaging data is required. Currently, the most commonly used methods conclude resampling and intensity normalization. Image resampling is used to improve image quality and eliminate bias introduced by non‐uniform imaging resolution.^49^, ^50^ Image intensity normalization is utilized to correct inter‐subject intensity variation by transforming all images from original greyscale into a standard greyscale.^51^, ^52^ Park et al normalized liver signal intensity according to the spleen signal on hepatobiliary phase (HBP) images to extract high‐order textural features and revealed the improved diagnostic value as compared with non‐normalized data.^29^

In addition to imaging data, clinical factors were also involved in radiomics analysis, including patient age, gender, Child‐Pugh stage, histologic grading, BCLC stage, cirrhosis and its cause, etc.^13^, ^14^, ^15^, ^16^, ^17^, ^18^, ^19^, ^20^, ^21^, ^22^, ^23^, ^24^, ^25^, ^26^, ^27^, ^28^, ^29^, ^30^, ^31^, ^32^, ^33^, ^34^, ^35^, ^36^, ^37^, ^38^, ^39^, ^40^, ^41^, ^42^, ^43^, ^44^, ^45^, ^46^, ^47^, ^48^ Laboratory examination indexes comprise serum α‐fetoprotein (AFP) level, prothrombin induced by vitamin K absence‐II (PIVKA‐II) level, carbohydrate antigen 19‐9 (CA 19‐9) level, hepatitis B virus surface antigen (HBsAg), serum alanine aminotransferase (ALT), aspartate aminotransferase (AST), serum total bilirubin (TB), conjugated bilirubin (CB), serum albumin (ALB), prothrombin time (PT), platelet count (PLT), etc.^13^, ^14^, ^15^, ^16^, ^17^, ^18^, ^19^, ^20^, ^21^, ^22^, ^23^, ^24^, ^25^, ^26^, ^27^, ^28^, ^29^, ^30^, ^31^, ^32^, ^33^, ^34^, ^35^, ^36^, ^37^, ^38^, ^39^, ^40^, ^41^, ^42^, ^43^, ^44^, ^45^, ^46^, ^47^, ^48^

### 2.2 Region of interest segmentation

Segmentation of region of interest (ROI) could be divided into manual segmentation and semiautomatic/automatic segmentation. Most radiomics studies on liver disease applied manual segmentation. Only six studies performed semiautomatic/automatic segmentation.^17^, ^30^, ^39^, ^46^, ^53^, ^54^

Manual segmentation is performed by radiologists to annotate the location and precise boundary of the lesion. Another way of manual segmentation is realized by placing a rectangular/circle box via deep learning analysis. Wang et al conducted a squared ROI segmentation as the input of convolution neural network (CNN) and achieved satisfying performance in liver fibrosis stage prediction.^16^ Naganawa et al applied similar segmentation approach with a 2‐cm diameter circular ROI covering the lesion while excluding intrahepatic vessels.^15^ Considering the discrepancy of subjective judgement in manual segmentation, segmentations by multi‐clinicians, of multi‐time point, and using computer perturbation are required to decrease the intra‐ and inter‐reader variability.^32^ Feature reproducibility and robustness are generally evaluated through calculation of intra‐class correlation coefficient and concordance correlation coefficient.^36^, ^56^, ^57^

Automatic segmentation aims to annotate ROIs by computer automatically, whereas semiautomatic segmentation still needs partial manual intervention to mark the centre of the lesion before automatic segmentation. Several classic segmentation algorithms showed good performance in liver lesion annotation.^58^, ^59^, ^60^, ^61^ These methods can be generally divided into three categories: (a) algorithms based on intensity thresholds and region (global thresholding, local thresholding, region growing, and region splitting and merging methods), (b) algorithms based on statistical approach (statistical parametric mapping and maximization segmentation algorithm), clustering (k‐means clustering and fuzzy clustering) and deformable model approach (Snake model and geometric active contour model), (c) algorithms incorporating empirical knowledge into the segmentation process (Atlas Guided Approach and Artificial Neural Network).

### 2.3 Feature extraction

Radiomic features are divided into manual engineered features and deep learning (DL) features. Manual engineered features include shape/histogram/texture‐based features. Shape‐based features describe the geometric attributes of the ROIs. Histogram features capture the first‐order statistic characteristics of liver parenchyma or liver lesion. Textural features, extracted from a series of high‐order textural matrixes, describe the granular textural pattern of the ROIs. In addition, filtered features are extracted from ROI preprocessed by wavelet, Laplacian and Gaussian filters from multiple dimensions.^62^ Commonly used manual engineered features are shown in Table 2. Another type of engineered features is defined as empirical features or semantic features that are designed by experience and knowledge of radiologists. Fu et al designed “peer‐off” features with hypothesis that tumour grows from inside to outside.^63^ By splitting the tumour into 10 peel‐off layers and extracting corresponding statistical features and its ratio, it can reflect tumour growth pattern and spatial heterogeneity. They found the feature ‐ POF_entropy showed satisfactory value for predicting the progress‐free survival following liver resection and transarterial chemoembolization. This feature exactly represented the texture randomness or irregularity of the innermost layer.

**TABLE 2:** Radiomic features used in radiomics studies on liver diseases

	Shape‐based 3D features (n = 16)	Shape‐based 2D features (n = 16)	Histogram features (n = 19)	Textural features (n = 75)				
				Gray Level Co‐occurrence Matrix (GLCM) Features (n = 24)	Gray Level Run Length Matrix (GLRLM) Features (n = 16)	Gray Level Size Zone Matrix (GLSZM) Features (n = 16)	Neighbouring Gray Tone Difference Matrix (NGTDM) Features (n = 5)	Gray Level Dependence Matrix (GLDM) Features (n = 14)
1	Mesh Volume	Mesh Surface	Energy	Autocorrelation	Short Run Emphasis (SRE)	Small Area Emphasis (SAE)	Coarseness	Small Dependence Emphasis (SDE)
2	Voxel Volume	Pixel Surface	Total Energy	Joint Average	Long Run Emphasis (LRE)	Large Area Emphasis (LAE)	Contrast	Large Dependence Emphasis (LDE)
3	Surface Area	Perimeter	Entropy	Cluster Prominence	Gray Level Non‐Uniformity (GLN)	Gray Level Non‐Uniformity (GLN)	Busyness	Gray Level Non‐Uniformity (GLN)
4	Surface Area to Volume ratio	Perimeter to Surface ratio	Minimum	Cluster Shade	Gray Level Non‐Uniformity Normalized (GLNN)	Gray Level Non‐Uniformity Normalized (GLNN)	Complexity	Dependence Non‐Uniformity (DN)
5	Sphericity	Sphericity	10th percentile	Cluster Tendency	Run Length Non‐Uniformity (RLN)	Size‐Zone Non‐Uniformity (SZN)	Strength	Dependence Non‐Uniformity Normalized (DNN)
6	Compactness	Spherical Disproportion	90th percentile	Contrast	Run Length Non‐Uniformity Normalized (RLNN)	Size‐Zone Non‐Uniformity Normalized (SZNN)		Gray Level Variance (GLV)
7	Spherical Disproportion	Maximum 2D diameter	Maximum	Correlation	Run Percentage (RP)	Zone Percentage (ZP)		Dependence Variance (DV)
8	Maximum 3D diameter	Major Axis Length	Mean	Difference Average	Gray Level Variance (GLV)	Gray Level Variance (GLV)		Dependence Entropy (DE)
9	Maximum 2D diameter (Slice)	Minor Axis Length	Median	Difference Entropy	Run Variance (RV)	Zone Variance (ZV)		Low Gray Level Emphasis (LGLE)
10	Maximum 2D diameter (Column)	Elongation	Interquartile Range	Difference Variance	Run Entropy (RE)	Zone Entropy (ZE)		High Gray Level Emphasis (HGLE)
11	Maximum 2D diameter (Row)		Range	Joint Energy	Low Gray Level Run Emphasis (LGLRE)	Low Gray Level Zone Emphasis (LGLZE)		Small Dependence Low Gray Level Emphasis (SDLGLE)
12	Major Axis Length		Mean Absolute Deviation (MAD)	Joint Entropy	High Gray Level Run Emphasis (HGLRE)	High Gray Level Zone Emphasis (HGLZE)		Small Dependence High Gray Level Emphasis (SDHGLE)
13	Minor Axis Length		Robust Mean Absolute Deviation (rMAD)	Informational Measure of Correlation (IMC) 1	Short Run Low Gray Level Emphasis (SRLGLE)	Small Area Low Gray Level Emphasis (SALGLE)		Large Dependence Low Gray Level Emphasis (LDLGLE)
14	Least Axis Length		Root Mean Squared (RMS)	Informational Measure of Correlation (IMC) 2	Short Run High Gray Level Emphasis (SRHGLE)	Small Area High Gray Level Emphasis (SAHGLE)		Large Dependence High Gray Level Emphasis (LDHGLE)
15	Elongation		Standard Deviation	Inverse Difference Moment (IDM)	Long Run Low Gray Level Emphasis (LRLGLE)	Large Area Low Gray Level Emphasis (LALGLE)		
16	Flatness		Skewness	Maximal Correlation Coefficient (MCC)	Long Run High Gray Level Emphasis (LRHGLE)	Large Area High Gray Level Emphasis (LAHGLE)		
17			Kurtosis	Inverse Difference Moment Normalized (IDMN)				
18			Variance	Inverse Difference (ID)				
19			Uniformity	Inverse Difference Normalized (IDN)				
20				Inverse Variance				
21				Maximum Probability				
22				Sum Average				
23				Sum Entropy				
24				Sum of Squares				

Filtered features extracted from images preprocessed by wavelet filter, Laplacian of Gaussian filter, etc, including the shape/histogram/texture‐based radiomic features.^63^

Compared with manual engineered features, DL network could extract supplementary high‐dimensional features that are hard to depict by observers.^55^, ^64^, ^65^, ^66^ The DL network encodes medical image into shape information and abstract textural information via shallow and deep layers respectively. Wang et al proposed a novel method to automatically extract DL features from MR imaging using CNN.^64^ They found that DL features outperformed textural features in predicting the malignancy of HCC. Chaudhary et al used unsupervised auto‐encoder framework to extract DL features.^66^ Features extracted from the bottleneck layer showed predictive ability for the survival risk of liver cancer.

### 2.4 Task‐oriented modelling

Generally, the methods for feature selection conclude filter‐based, wrapper‐based, and model‐embedded methods.^67^ Filter‐based methods produce a selected feature set according to the correlation between features and the classifying labels. Commonly used filter‐based methods include calculation of mutual information, correlation coefficient and uni‐variable analysis (ie Mann‐Whitney U test and Chi‐squared test), etc.^68^, ^69^, ^70^ Wrapper‐based methods take into account the weighing of feature subsets, and are combined with an appointed classifier. It selects features that could improve the accuracy of the prediction to the maximum extend and removes the features that contribute less to the prediction until the specified feature number is reached. Model‐embedded methods perform feature selection in the process of model construction. An example of this method is the least absolute shrinkage and selection operator (LASSO) algorithm.^71^ LASSO aims to minimize the residual sum of squares, subjected to the sum of the absolute value of the coefficients being less than a tuning parameter. It forces specified coefficients to zero and thus effectively produce a simpler model. Among the aforementioned methods, filter‐based methods require less computation time than the other two methods but with lower prediction accuracy. Thus, they are most commonly used as a primary selection method to initially reduce features.^23^, ^55^

Regarding modelling strategy, radiomics studies on liver disease mostly utilized supervised learning modelling. LASSO logistic regressing modelling was commonly used, demonstrating satisfying performance particularly in small sample size based studies.^22^, ^31^, ^72^ Support vector machine and random forest were also used in published liver disease radiomics studies.^19^, ^23^, ^27^, ^32^ Notably, Li et al compared six types of machine‐learning algorithms in predicting liver fibrosis, including adaptive boosting, decision tree, logistic regression, neural network, random forest and support vector machine.^20^ Their result indicated that adaptive boosting, random forest and support vector machine stood out as superior modelling methods with improved accuracy for fibrosis prediction.

## 3 RADIOMICS IN THE DIAGNOSIS AND STAGING OF LIVER DISEASES

**FIGURE 2:**
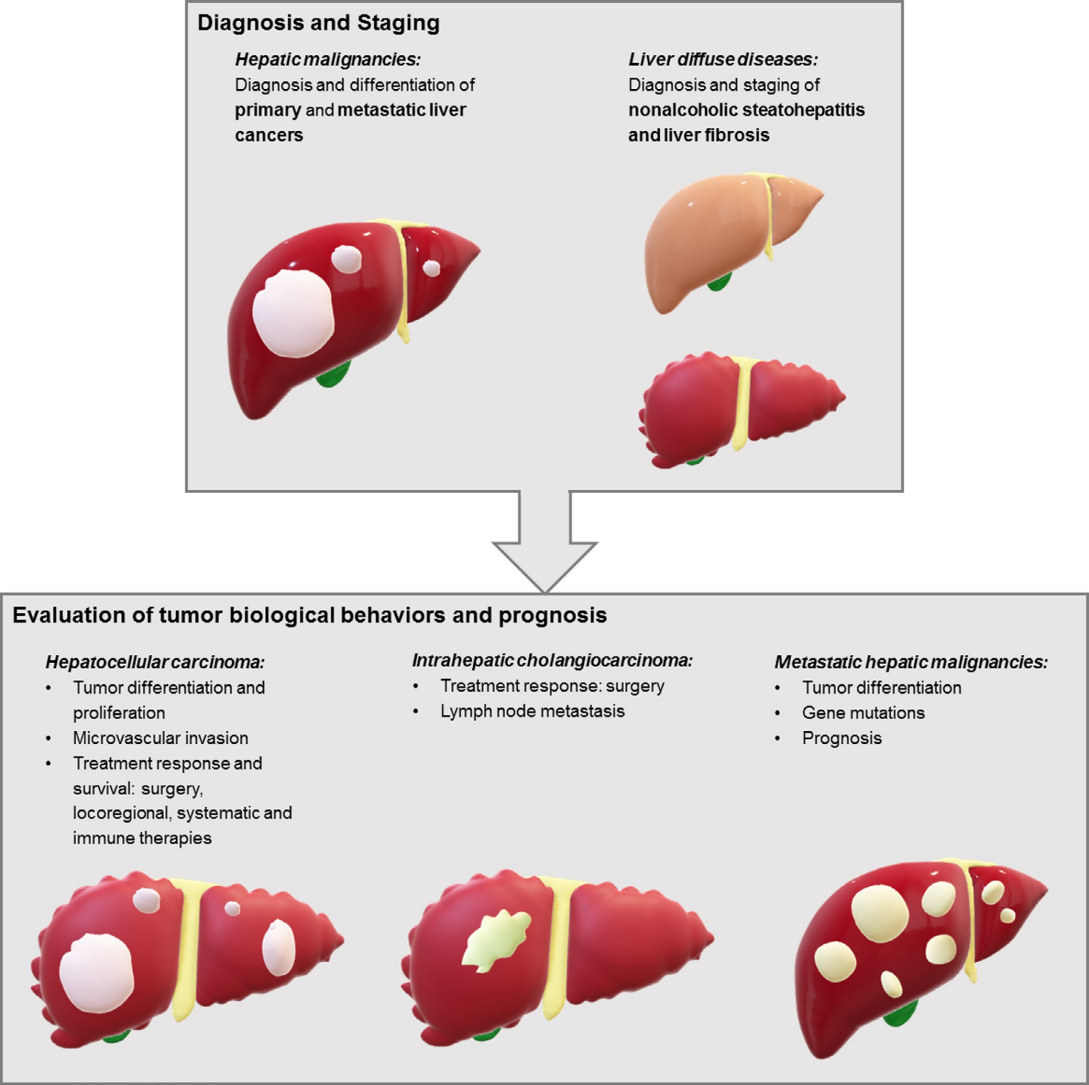
Illustration of clinical application of radiomics on liver diseases

For clinical application, radiomics plays a pivotal role in the diagnosis, staging and grading of several liver diseases, of which most efforts focused on hepatic malignancies and liver diffuse diseases (Figure 2).

### 3.1 Hepatic malignancies

Hepatocellular carcinoma (HCC) is currently the most common primary liver cancer.^73^ However, many non‐HCC malignancies (eg small duct type intrahepatic cholangiocarcinoma [ICC] and combined hepatocellular‐cholangiocarcinoma) and other atypical benign focal liver lesions (eg haemangioma and hepatic adenoma) can mimic HCC, making the diagnosis challenging via current imaging techniques.^74^, ^75^

Radiomics demonstrated great potential in differentiating focal liver lesions.^25^, ^76^, ^77^ Li et al primarily investigated texture features of focal hepatic lesions on spectral attenuated inversion‐recovery T2 weighted MRI, and found that the radiomics signatures can help classify hepatic haemangioma, hepatic metastases and HCC with satisfying diagnostic performances (area under the curve [AUC]: 0.83‐0.91).^76^ Trivizakis et al reported that the three‐dimensional convolutional neural network features on diffusion‐weighted MR images achieved an accuracy of 83% for discriminating primary and metastatic liver tumours.^77^ In addition to MR imaging, radiomics analysis on multi‐modal ultrasound images also demonstrated diagnostic ability for benign and malignant focal liver lesion classification (AUC: 0.94, 95%CI: 0.88‐0.98) and malignant subtyping (AUC: 0.97, 95%CI: 0.93‐0.99).^25^

### 3.2 Liver diffuse diseases

Besides hepatic malignancies, radiomics also showed potential in characterization of liver diffuse diseases including fatty liver diseases and liver fibrosis. The first study evaluating the performance of CT‐based texture features for predicting nonalcoholic steatohepatitis (NASH) was conducted by Naganawa et al, which included 88 retrospective suspected NASH patients.^15^ They reported that the AUC reached up to 0.94 in patients without suspected fibrosis, but dropped significantly in patients with suspicion of fibrosis (AUC: 0.60). Tang et al further explored the relationship between a quantitative ultrasound‐based machine learning model and histopathology scoring in a rat model.^78^ Their results demonstrated that combining quantitative ultrasound parameters with conventional shear wave elastography significantly improved the classification accuracy of steatohepatitis, liver steatosis, inflammation and fibrosis.

Other than fatty liver diseases, more studies focused on liver fibrosis staging and associated complications. A prospective multi‐centre study by Wang et al revealed that DL radiomics of shear wave elastography (SWE) significantly improved the accuracy of liver fibrosis staging, with AUCs of 0.97, 0.98 and 0.85 for cirrhosis (F4), advanced fibrosis (≥F3) and significant fibrosis (≥F2) respectively.^16^ Similar results have been reported by another prospective study, in which the machine‐learning‐based multi‐parametric ultrasomics model achieved remarkably improved power for significant fibrosis (≥F2).^20^

CT‐based radiomics was also utilized for noninvasive assessment of liver fibrosis. Choi et al retrospectively developed a DL system on portal venous phase CT images in 7461 patients and validated it in an independent data sets comprising 891 patients.^79^ The accuracy was of 79.4% in the validation sets, with AUC of 0.96, 0.97 and 0.95 for ≥ F2, ≥F3 and F4 respectively. Regarding portal hypertension, Liu et al reported in their multi‐centre prospective study that the radiomics signature on portal venous phase CT images accurately detected portal hypertension with the C‐index of 0.889, 0.800, 0.917 and 0.827 in four external validation cohorts respectively.^23^

## 4 RADIOMICS IN THE EVALUATION OF LIVER TUMOUR BIOLOGICAL BEHAVIOURS AND PROGNOSIS

Beyond diagnosis and staging, radiomics enables quantitative assessment of liver tumour biological behaviours, as well as prediction of prognosis and antitumoral treatment effect (Figure 2).

### 4.1 HCC

#### 4.1.1 Measurement of tumour differentiation and proliferation

Histologic grade was one of the most important risk factors for postoperative recurrence in HCC.^80^, ^81^, ^82^, ^83^ Recently, two MRI‐based studies investigated radiomic features for HCC aggressiveness characterization, demonstrating the potential of radiomics as indicative biomarkers for HCC grade.^24^, ^84^ Regarding Ki‐67 level, Ye et al reported that radiomics analysis can evaluate the tumour Ki‐67 level preoperatively with good accuracy (C‐index: 0.936) in a prospective study.^85^

#### 4.1.2 Assessment of tumour vascular invasion

Preoperative discrimination between neoplastic and bland portal vein thrombosis and detection of microvascular invasion in HCC is critically important.^86^, ^87^ Canellas et al explored the role of CT texture features for differentiating neoplastic and bland portal vein thrombosis. They found that mean value of positive pixels and entropy can characterize portal vein thrombosis.^88^ Recent studies have shown promising results of CT and ultrasound‐based radiomics signatures for preoperative microvascular invasion prediction, all with high diagnostic accuracy.^17^, ^89^

#### 4.1.3 Prediction of treatment efficacy and prognosis

Radiomics analysis permits accurate prediction of prognosis and effective diverse therapy evaluation.^73^, ^90^ Several studies were conducted for hepatic resection evaluation, and one study was for liver transplantation evaluation.^13^, ^19^, ^21^, ^28^, ^91^, ^92^, ^93^ Furthermore, Li et al found that texture analysis of CT images can be helpful not only in prognosis prediction, but also in treatment selection between liver resection and transcatheter arterial chemoembolization (TACE).^81^ For HCC patients with prominent vascular invasion and/or extrahepatic spread (BCLC stage C), systematic treatment is the standard of care recommended by current guidelines from different geographical regions.^36^, ^90^ Mulé et al retrospectively investigated 92 advanced HCC patients from two centres and reported that the contrast‐enhanced CT texture feature entropy was correlated with tumour heterogeneity by manual visualization, and entropy on portal venous phase images was an independent predictor for OS.^94^

Radiomics analysis also yielded promising results in predicting response for patients treated with immunotherapies. Sun et al retrospectively generated a contrast‐enhanced CT‐based radiomics signature of tumour‐infiltrating CD8 cells and investigated its performances in predicting tumour immune phenotype (immune‐inflamed vs immune‐desert) and response to anti‐programmed cell death protein (PD)‐1 or anti‐programmed cell death ligand 1 (PD‐L1) monotherapies.^95^ Another study by Chen et al explored the capacity of radiomics analysis on gadoxetic acid‐enhanced MR imaging in predicting immunoscore, a new prognostic biomarker for immunotherapy revealing tumour infiltrating lymphocytes density.^96^

### 4.2 ICC

ICC is an aggressive primary hepatic cancer arising from the bile duct epithelium.^97^ However, unlike HCC, surgical resection is currently the only curative treatment for ICC patients.^98^ A recent single‐centre retrospective study reported that the radiomics signature on preoperative arterial‐phase contrast‐enhanced MR images can be used to predict early recurrence of ICC after partial hepatectomy with the AUC of 0.82 and 0.77 in the training and validation cohort respectively.^55^ Ji et al constructed a radiomics signature from portal venous CT to predict lymph node metastasis in biliary tract caners.^99^ They found good discrimination of the signature in both training (AUC: 0.81) and validation cohort (AUC: 0.80).^99^

### 4.3 Metastatic hepatic malignancies

In addition to primary liver cancers, radiomics also showed promise in the evaluation of several metastatic hepatic malignancies. Lubner et al found that pretreatment portal venous phase CT texture features of the colorectal liver metastases were significantly associated with tumour grade, KRAS mutation and OS.^100^ Another retrospective study investigated the ratio between the texture of colorectal liver metastases and the surrounding liver, and found that it may reflect tumour aggressiveness, chemotherapy response and OS.^101^ However, Lee et al reported that texture features from liver parenchyma on portal venous phase CT cannot be used to predict the development of hepatic metastasis in colorectal cancer patients.^102^ Apart from colorectal cancer, emerging evidence suggests that the CT‐based radiomics signature of esophagogastric liver metastases can help predict treatment response to chemotherapy.^27^

## 5 FUTURE CHALLENGES AND OPPORTUNITIES

Current published studies revealed the potential of radiomics analysis in liver disease diagnosis, tumour biological property profiling, and prognosis estimation. However, although MR imaging can provide the multi‐parametric information regarding hepatic function and microenvironment with higher tissue resolution, most studies to date have focused on radiomics analyses of CT.^103^, ^104^, ^105^, ^106^ In addition, a large number of studies were retrospective in design and lack independent external validation across different geographical areas and races, which may limit the generalizability and applicability of the current findings. Different prevalence of disease may also influence the accuracy of the algorithm (*eg* positive and negative predictive values). Moreover radiomics results are extremely sensitive to the various technical acquisition parameters, especially among different vendors. Therefore, more large scale multi‐centre prospective studies with standardized acquisition, segmentation and imaging postprocessing are needed to ensure further development of radiomics in liver diseases.

## 6 CONCLUSIONS

Radiomics as a newly emerged quantitative technique is burgeoning in liver disease management with consistently developing methodology. Previous studies, although mainly retrospective in design and based on single imaging modality, have revealed its potential in diagnosis, treatment evaluation and prognosis prediction of several liver diseases. Nevertheless, further multi‐centre and prospective validation is still needed to valid its clinical usefulness, especially in prognosis‐related targets.

Current main obstacles for the application of radiomics in liver disease rely on high‐quality data collection and mechanism explanation on the biological basis. Multi‐institutional data sharing and intensive collaborations on data cleansing and labelling offer appeal in filling this gap. Artificial intelligence algorithms with improved accuracy and interpretability meanwhile need to be developed to facilitate broader translation and clinical adoption.

## 7 Financial information

This study has received funding by Ministry of Science and Technology of China under Grant No. 2017YFA0205200, National Natural Science Foundation of China under Grant No. 81227901 and 81527805, Chinese Academy of Sciences under Grant No. GJJSTD20170004 and QYZDJ‐SSW‐JSC005, Beijing Municipal Science & Technology Commission under Grant No. Z161100002616022 and 171100000117023.

## CONFLICT OF INTEREST

None.
